# Research progress of E3 ubiquitin ligase regulating biological behavior of human placental trophoblast cells

**DOI:** 10.3389/fendo.2023.1124041

**Published:** 2023-04-14

**Authors:** Jun Feng, Huimei Yin, Baturuhu, Yonghui Dai, Fuxiao Dai, Junjun Xu, Zhili Chen, Yanyan Liu

**Affiliations:** ^1^Department of Emergency Medicine, Tongji Hospital, Huazhong University of Science and Technology, Wuhan, China; ^2^Department of Emergency Medicine, People’s Hospital of Bortala Mongol Autonomous Prefecture, Bole, China; ^3^Department of Neurosurgery Intensive Care Unit (ICU), People’s Hospital of Bortala Mongol Autonomous Prefecture, Bole, China; ^4^Obstetrics Department, People’s Hospital of Bortala Mongol Autonomous Prefecture, Bole, China; ^5^Obstetrics and Gynecology Department, Tongji Hospital, Huazhong University of Science and Technology, Wuhan, China

**Keywords:** E3 ubiquitin ligase, human trophoblast cells, *in vitro* migration, invasiveness, apoptosis, proliferation and differentiation, pregnancy complications

## Abstract

E3 ubiquitin ligases are important components of the ubiquitin protease system. This family includes many proteins, which can catalyze the ubiquitination of a variety of protein substrates and promote the degradation of them by the proteasome system. Recent studies have shown that E3 ubiquitin ligase plays a key role in the process of fetal development and placental formation. It affects the biological behavior of placental trophoblast cells, leading to a series of pregnancy complications that threaten mothers and babies greatly. This review focuses on the regulation, target and mechanism of E3 ubiquitin ligase on the biological behavior of human placental trophoblast cells.

## 1 Introduction

The formation of human placenta begins with embryo implantation, involving the invasion of trophoblastic cells into the uterine epithelium and matrix. It is the result of the interaction between maternal and fetal tissues, including a series of complex processes of invasion, migration, proliferation and differentiation of trophoblastic cells. The abnormal biological behavior of trophoblast cells is closely related to the occurrence of many pregnancy diseases. Inadequate trophoblastic invasion in early pregnancy is related to pathological pregnancy such as preeclampsia (PE), fetal growth restriction (FGR), recurrent spontaneous abortion (RSA) (1–3), and its excessive invasion is related to hydatidiform mole and choriocarcinoma (4). The precise regulation was related to complex gene expression regulation mechanisms and protein interactions.

In mammals, protein degradation is mainly regulated by lysosomal pathway and ubiquitin proteasome pathway to maintain homeostasis and normal cell function. Among them, the lysosomal pathway mainly plays a role in the stress state, while the degradation of short-lived proteins in the body mainly depends on the ubiquitin proteasome pathway, through which 80% of proteins are degraded. This pathway is not only important for maintaining the dynamic balance of proteins in the body (5), but also can participate in the regulation of cell cycle, apoptosis, inflammatory response, oxidative stress response, gene transcription, signal transduction (6) and other important processes. At present, studies have shown that E3 ubiquitin ligase plays an important role in the ubiquitin proteasome degradation pathway, as well as in the formation and function of human placenta. This article summarized the research progress of biological behavior of E3 ubiquitin ligase in human placental trophoblast cells in recent years, aiming to provide a strong scientific basis for further exploring the molecular mechanism, target and regulatory mechanism of pregnancy complications.

## 2 E3 ubiquitin ligase regulates invasion and migration of human placental trophoblasts

### 2.1 Cullin protein family

#### 2.1.1 Cullin1

The study on the role of CUL1 in trophoblast differentiation during placental development is still in its infancy (2, 5, 7). Immunohistochemistry confirmed the expression of CUL1 in CTB cells and EVT of human placenta in early pregnancy. Q Zhang’s research shows that CUL1 siRNA significantly inhibited outgrowth of extravillous explants *in vitro*, as well as invasion and migration of HTR8/SVneo cells of EVT origin, exogenous CUL1 promoted invasion and migration of HTR8/SVneo cell (7). CUL1 played a role in promoting the infiltration and migration of human trophoblast cell line, and detected the down-regulation of CUL1 in trophoblast cells of patients with PE, a pregnancy disease that poses a great threat to the mothers, which is consistent with the phenomenon of insufficient infiltration of trophoblast cells that may cause PE (8).

#### 2.1.2 Cullin3

Most Cullin proteins play a key role in embryonic development, and Cullin 3 is a major member of the CUL protein family. More and more experiments have proved that the BCR formed by CUL3 plays an important role in cell cycle regulation and ontogeny. In living villus tissue, the ability of trophoblast cells at the top of villus to migrate outward will be significantly inhibited by adding small RNA interfering with the expression of CUL3 to the explant culture model (9). At the same time, the same interference experiment was also carried out in the cell line HTR8/SVneo derived from human placental extravillous trophoblast cells, and affected the migration and invasion ability of HTR8/SVneo cells *in vitro* (10). Further studies showed that the interference of CUL3 was accompanied by the decrease of the activity of matrix metalloproteinase 9 precursor (pro-MMP9) and the up regulation of tissue inhibitors of metalloproteinase 1 and 2 (TIMP1/2) (11).

One of the pathological features of PE is superficial invasion of endometrium by trophoblast cells. Immunohistochemical staining of CUL3 on pathological sections of the disease samples and normal placental tissues of the same gestational weeks also found that the low expression of CUL3 was related to the low migration and invasion ability of these trophoblastic cells (12). Using lentivirus mediated placental specific gene knockout technique, a mouse model with placental specific knockdown of CUL3 expression was constructed. By analyzing the placental structure of the knockdown CUL3, it was found that the number of glycogen trophoblast cells, a type of trophoblast cells that infiltrated the decidua of the mother, would be significantly reduced (13). The above results indicate that CUL3 can regulate the invasion and migration of trophoblast cells, and the disorder of its expression and regulation may also lead to the occurrence of PE (9).

One possible explanation is that hypoxia stimulates the production of large amounts of HIF-1 α, HIF-1 α Accumulation increases the expression of speckle-type POZ protein(SPOP) in trophoblast (14). As the connector protein of cullin3, SPOP targets PI3K/AKT/GSK3 and damage the mobility of trophoblast (15). This may lead to inadequate remodeling of the uterine spiral artery and inadequate placental perfusion, leading to pregnancy related complications.

### 2.2 F-box and WD repeat domain-containing 8

Fbxw8 is the F-box protein in the ubiquitin E3 ligase complex, which mainly plays the role of recognizing the substrate to be degraded. When Fbxw8 is knocked out, the mice show placental development defects and embryonic development retardation. Fbxw8 is widely expressed in early pregnancy placental villi and trophoblast cells derived from early placental villi, suggesting that Fbxw8 may play an important role in early pregnancy (16). Targeted inhibition of Fbxw8 expression can inhibit the invasion and migration of trophoblast cells by reducing the activities of MMP-2 and MMP-9. The effect of Fbxw8 on JEG-3 cell proliferation was negatively correlated with the expression of cyclin dependent kinase inhibitor p27, and positively correlated with the expression of G2/M cell cycle regulatory proteins CDK1, CDK2, cyclin A and cyclin B1 (17); Further studies showed that this effect may be achieved by regulating the expression of anti-proliferation gene BTG2 (18).

### 2.3 Casitas B-cell lymphoma

Cbl protein family is a group of E3 ubiquitin ligases with RING (Really Interesting New Gene) finger domain (19–21). The expression of Cbl in placental trophoblasts of early severe PE was down-regulated. In HTR8/SVneo cell model, hypoxia stimulation can significantly increase the signal of Met cluster aggregation in cells and reduce the expression of Cbl; At the same time, the percentage of co localization of Met and proteasome in cells also decreased significantly. Knockdown of Cbl expression in cells can increase Met cluster like aggregation signal and decrease the co localization percentage of Met and proteasome, suggesting that the degradation of Met proteasome pathway is weakened. When the expression of Cbl was knocked down in cells, HGF almost completely blocked the activation of Met and Erk and the promotion of trophoblast invasion (22). These results suggest that the Cbl mediated degradation of Met ubiquitination is critical to the function of HGF/Met signaling, and hypoxia stimulation can lead to the down-regulation of Cbl expression, so the degradation of Met ubiquitination is blocked, a large number of Met proteins are accumulated in the cytoplasm, and the function of HGF to regulate the behavior of trophoblast cells cannot be realized (19–22).

### 2.4 β-transducin repeat containing protein

β- TrCP is a substrate recognition subunit of E3 ubiquitin ligase, which can specifically recognize ubiquitinated substrates and play an important role in cell proliferation, signal transduction and cell cycle process (23). Abnormality of β-TrCP protein expression or dysfunction often leads to abnormal ubiquitination modification, which affects the occurrence and development of many diseases.

Cell experiments showed that overexpression of miR-135a-5p could promote the migration and invasion of trophoblast cells *in vitro*, β- TrCP has been proved to be the target gene of miR-135a-5p in trophoblast (24). Molecular experiments showed that overexpression of miR-135a-5p could induce the decrease of E-cadherin level and N-cadherin, Vimentin and β-catenin level increased, while β-TrCP overexpression will weaken this effect (23, 24). In conclusion, more and more studies show that miR-135a-5p passes the target β- TrCP promotes the migration and invasion of trophoblast cells.

Research has confirmed that small molecule inhibitor PDTC can inhibit β- TrCP, and exert anti-tumor effect (25). Therefore, in-depth research on β-TrCP can further understand the gestational diseases caused by trophoblastic abnormalities, and help to provide new ideas and potential therapeutic targets for clinical treatment of pregnancy related complications.

### 2.5 Smad ubiquitin regulatory factor 2

Smurf2 is an E3 ubiquitin ligase that participates in Smad-mediated TGF-β Signal conduction, which plays an important role in the normal embryo implantation process, but whether Smurf2 participates in this process has not been reported (26). The study on the expression of Smurf2 in different parts of the uterus and placenta of rhesus monkeys in early pregnancy showed that Smurf2 may regulate TGF during early pregnancy, the expression of TGF-β signal pathway related proteins plays a specific role in gland secretion, trophoblast invasion and placental formation (26, 27).

## 3 E3 ubiquitin ligase regulates apoptosis of human placental trophoblasts

### 3.1 Mcl-1 ubiquitin ligases E3

The E3 ubiquitin ligase MULE targets myeloid cell leukemia factor 1 (Mcl-1) and tumor suppressor p53 for proteasomal degradation. Rolfo A’s research (28) shows that Mcl-1 and p53 are related to trophoblast cell death in PE and FGR, and regulate their ubiquitination during placental development, further research shows that MULE is overexpressed in both PE and FGR placentas, MULE preferentially targets p53 degradation in PE, allowing the accumulation of apoptosis promoting Mcl-1 subtypes, however, in FGR, MULE targeted to promote the survival of Mcl-1, making p53 accumulate and play its apoptotic function, different priority targets of MULE in PE and FGR placenta also classify early-onset PE and FGF as different molecular pathology.

### 3.2 MDM2

MDM2 is an important gene that regulates p53 pathway, has the function of p53 degradation and ubiquitination, participates in the occurrence and development of tumors, and is related to embryonic development and tissue differentiation (29). Recent research shows that p53/Mdm2 system can mediate β-arrestin1 expression, which plays an important role in maintaining maternal-fetal tolerance, the decreased expression of β-arrestin1 in the villous samples may be related with the development of missed abortion (30).

## 4 E3 ubiquitin ligase regulates proliferation and differentiation of human placental trophoblasts

### 4.1 Fbxw8

The effect of Fbxw8 on JEG-3 cell proliferation was negatively correlated with the expression of cyclin dependent kinase inhibitor p27, and positively correlated with the expression of G2/M cell cycle regulatory proteins CDK1, CDK2, cyclin A and cyclin B1; Further studies showed that this effect may be achieved by regulating the expression of anti-proliferation gene BTG2 (17, 18).

### 4.2 Cullin7

Recent research shows that CUL7 E3 ligase is a key regulator in trophoblast cell epithelial-mesenchymal transition and placental development, but its specific mechanism still needs to be further studied (31).

## 5 Outlook

In the process of the occurrence and development of obstetric diseases, protein, as an important executor of gene function, has important significance in the dynamic balance of the body. E3 ubiquitin ligase plays an important role in the degradation of proteins by the ubiquitin proteasome pathway. The current research focuses on the relationship between the degradation of specific substrates by E3 ubiquitin ligase and its expression level and clinical prognosis. In recent years, a large number of studies have shown that E3 ubiquitin ligase is related to the occurrence and development of a series of obstetric diseases. CUL1 and CUL3 can regulate the invasion and migration of trophoblast cells in PE (7–9, 13); The abnormal weakening of cbl mediated Met signaling pathway inhibits the invasion and migration of trophoblasts (20, 22); E3 ubiquitin ligase MULE targets myeloid leukemia factor 1 (Mcl-2) in FGR (28), making p53 accumulate and play its apoptosis function (32); In RSA, abnormal Cullin1 ubiquitin deletion mediated p21 accumulation participates in the pathogenesis of RSA by regulating the proliferation and differentiation of trophoblasts (33).

Most studies focus on revealing the role of E3 ubiquitin ligase in tumors, but the study on the role of E3 ubiquitin ligase in human placental trophoblastic diseases is still insufficient. As a potential target for clinical treatment, E3 ubiquitin ligases still need to be further analyzed in the future. Finding the specific substrate of E3 ubiquitin ligase is also one of the difficulties faced at present. Adjusting the interaction between E3 ubiquitin ligase and its corresponding substrate may provide new ideas for personalized therapy, gene therapy drug development and clinical drug use.

## Author contributions

JF: writing original draft and editing. HY: writing original draft. B: review and editing. YD: writing-original draft and editing. FD: writing original draft. JX: writing original draft. ZC: writing original draft. YL: writing-review and editing and supervision and funding acquisition. All authors contributed to the article and approved the submitted version.
